# Impaired neurogenesis, learning and memory and low seizure threshold associated with loss of neural precursor cell survivin

**DOI:** 10.1186/1471-2202-11-2

**Published:** 2010-01-05

**Authors:** Vanessa Coremans, Tariq Ahmed, Detlef Balschun, Rudi D'Hooge, Astrid DeVriese, Jonathan Cremer, Flavia Antonucci, Michaël Moons, Veerle Baekelandt, Veerle Reumers, Harold Cremer, Amelia Eisch, Diane Lagace, Tom Janssens, Yuri Bozzi, Matteo Caleo, Edward M Conway

**Affiliations:** 1 KU Leuven, VIB Vesalius Research Center (VRC), Herestraat 49, Gasthuisberg O&N1, B3000 Leuven, Belgium; 2 KU Leuven Laboratory of Biological Psychology, Tiensestraat 102, B3000 Leuven, Belgium; 3 Dept of Pharmacology, University of Milan, via Vanvitelli 32, Milan, Italy; 4 Istituto di Neuroscienze, Consiglio Nazionale delle Ricerche, via G. Moruzzi 1, 56100 Pisa, Italy; 5 KU Leuven, Laboratory for Neurobiology and Gene Therapy, Kapucijnenvoer 33, B3000 Leuven, Belgium; 6 Developmental Biology Institute of Marseille, NMDA CNRS, INSERM, Univ. de Mediterranee, Campus de Luminy, 13288 Marseille, France; 7 Department of Psychiatry, University of Texas Southwestern Medical Center, 5323 Harry Hines Blvd, Dallas, Texas, 75390-9070 USA; 8 Department of Cellular and Molecular Medicine & Neuroscience Program, University of Ottawa, 451 Smyth Road, Ottawa, K1H 8M5 Canada; 9 Laboratory of Molecular Neuropathology, Centre for Integrative Biology, University of Trento, via delle Regole 101, 38060 Trento, Italy; 10 Center for Blood Research, Faculty of Medicine, University of British Columbia, 2350 Health Sciences Mall, Vancouver, V6T 1Z3 Canada

## Abstract

**Background:**

Survivin is a unique member of the inhibitor of apoptosis protein (IAP) family in that it exhibits antiapoptotic properties and also promotes the cell cycle and mediates mitosis as a chromosome passenger protein. Survivin is highly expressed in neural precursor cells in the brain, yet its function there has not been elucidated.

**Results:**

To examine the role of neural precursor cell survivin, we first showed that survivin is normally expressed in periventricular neurogenic regions in the embryo, becoming restricted postnatally to proliferating and migrating NPCs in the key neurogenic sites, the subventricular zone (SVZ) and the subgranular zone (SGZ). We then used a conditional gene inactivation strategy to delete the *survivin *gene prenatally in those neurogenic regions. Lack of embryonic NPC survivin results in viable, fertile mice (*Survivin*^*Camcre*^) with reduced numbers of SVZ NPCs, absent rostral migratory stream, and olfactory bulb hypoplasia. The phenotype can be partially rescued, as intracerebroventricular gene delivery of survivin during embryonic development increases olfactory bulb neurogenesis, detected postnatally. *Survivin*^*Camcre *^brains have fewer cortical inhibitory interneurons, contributing to enhanced sensitivity to seizures, and profound deficits in memory and learning.

**Conclusions:**

The findings highlight the critical role that survivin plays during neural development, deficiencies of which dramatically impact on postnatal neural function.

## Background

In the adult, two major, well-defined neurogenic regions persist [1]. In the subventricular zone (SVZ), neural precursor cells (NPCs) that arise mostly from the embryonic lateral ganglionic eminence (LGE) [2], continuously proliferate, and then migrate tangentially along the rostral migratory stream (RMS) towards the olfactory bulb (OB) where they differentiate into granular and periglomerular inhibitory interneurons [3]. In the subgranular zone (SGZ) of the hippocampus, newborn NPCs also migrate, but for shorter distances, into the granule cell layer, where they become excitatory granule cells [4]. From these neurogenic sites, adult-generated neurons can migrate to regions of brain injury [5], and establish synaptic contacts and functional connections [6,7]. Decreased neurogenesis induced by prenatal or postnatal stresses is implicated in the development of seizures and disorders in learning, memory and cognition [8,9]. The possibility of preventing onset or progression of these neural diseases by therapeutically enhancing neurogenesis [10-15] is prompting efforts to delineate the mechanisms and regulatory factors underlying NPC survival, proliferation, differentiation, migration and function. Indeed, numerous neuro-regulatory transcription factors, growth factors and receptors have been identified and characterized (reviewed in [16-19]). However, in spite of advances, effective approaches to prevent and treat diseases of the central nervous system are lacking, underlining the urgent need to develop better models to elucidate the molecular mechanisms regulating integration of new neurons in the developing and adult brain. Survivin is a member of the inhibitor of apoptotis protein (IAP) family, that also promotes the cell cycle and is a chromosome passenger protein [20,21]. During embryonic development, it is expressed by several tissues, but is particularly prominent in the nervous system [22]. Inactivation of the *survivin *gene in neuroepithelial cells early in development [23] results in massive apoptosis throughout the central nervous system, with total destruction of the architecture of the brain and lethality. The severity of this phenotype precluded investigators from delineating the specific role of neural precursor cell survivin on postnatal neural function.

Therefore, to elucidate the properties of survivin in neural development and function, we inactivated the *survivin *gene in NPCs in the late-midterm murine embryo and evaluated the effects post-natally. By this approach, we generated a unique *in vivo *mouse model in which reduced neurogenesis is associated with epilepsy and profound deficits in learning and memory. Pilot rescue studies suggest that embryonic administration of survivin may enhance neurogenesis. The findings highlight the critical role that survivin plays during neural development, deficiencies of which dramatically impact on postnatal neural function.

## Results

### Survivin is expressed in precursor cells in the neurogenic areas of the brain

**Figure 1:**
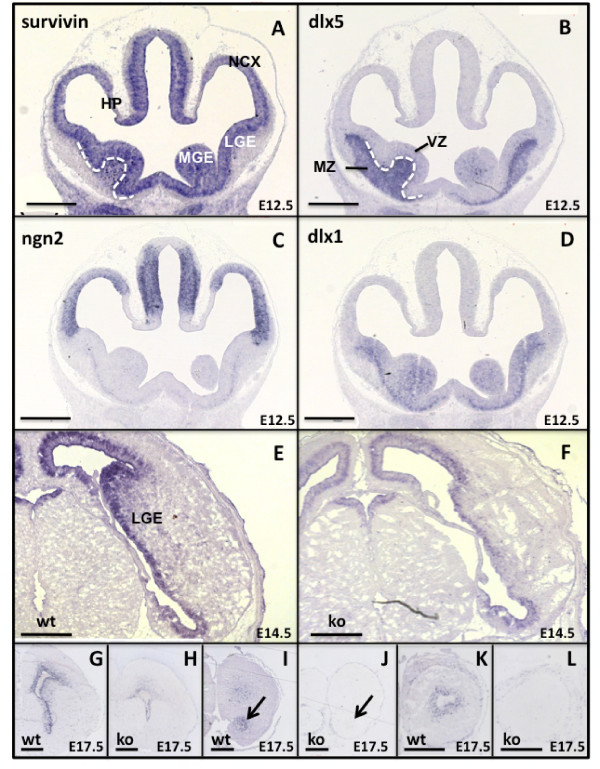
**Expression of survivin mRNA in developing mouse brain**. *In situ *hybridizations were performed on coronal brain sections from control (wt) (A-D, G, I, K) and *Survivin*^*Camcre *^(ko) (H, J, L) embryos, and on transverse brain sections from control (E) and *Survivin*^*Camcre *^(F) embryos. **(A-D) **mRNA expression of *survivin *(A), dlx5 (B), ngn2 (C), and dlx1 (D) on adjacent brain sections from control mice illustrates overlap of survivin expression with dlx1 and ngn2. Dashed white line (A, B) indicates minimal overlap of survivin with dlx5. **(E-L) **Expression of *survivin *mRNA is reduced in the medial and lateral ganglionic eminences (MGE, LGE) (F, H), the RMS (arrow, J), and the OB (L) in *Survivin*^*Camcre *^embryos as compared to controls. NCX, neocortex; HP, hippocampus. Scale bars: 500 μm

*In situ *hybridization using a probe against full length survivin allowed assessment of the spatiotemporal expression of survivin during embryonic development. *Survivin *mRNA was detected in the neurogenic areas of the dorsal and ventral telencephalon surrounding the ventricles (neocortex, medial and lateral ganglionic eminences (MGE and LGE, respectively)) (Figure 1A). At E12.5, expression of survivin overlapped with Dlx1, a marker for mitotic cells in the MGE and LGE [24] (Figure 1A, D) and neurogenin 2 (Ngn2) [25], a marker for dividing precursors in the neocortex (Figure 1A, C). There was minimal overlap with Dlx5 (Figure 1B), which is primarily expressed by postmitotic cells in the mantle zone of the MGE and LGE, less so in mitotic cells in the SVZ, and almost absent in the ventricular zone [26]. By E17.5, survivin was additionally expressed in the rostral migratory stream (RMS) and at the center of the olfactory bulb (OB) (Figure 1G, I, K). Survivin mRNA was also present in the retina and lens of the developing eye (not shown).

**Figure 2:**
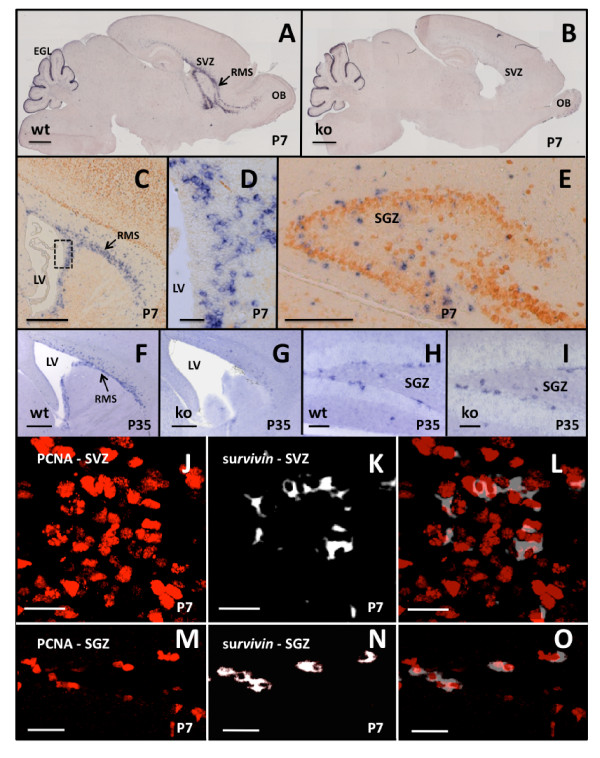
**Postnatal expression of survivin mRNA in neural precursor cells**. Sagittal sections of brains were used to examine expression of survivin. **(A, B) **At P7, *Survivin *mRNA was detected in the DG, SVZ, RMS, OB, and EGL of controls (A), and absent in the SVZ and RMS, but not in the DG and EGL of *Survivin*^*Camcre *^mouse brains (B). **(C-E) **Double labeling to detect *survivin *mRNA (blue) and NeuN protein (orange) expression in the SVZ-RMS (C, D) and the DG (E) in P7 control brains, shows that *survivin *is not expressed in NeuN+ cells. D is a magnified view of the dashed box in C. **(F-I) ***Survivin *mRNA expression at P35 is restricted to neural precursor cells in the SVZ, RMS and SGZ in control mice (F, H). *Survivin *expression is reduced in the SVZ and RMS (G), but not in the SGZ (I) of *Survivin*^*Camcre *^mice (ko) as compared to controls (wt). **(J-O) **Sagittal sections of control P7 brains through the SVZ (J-L) and through the SGZ (M-O) were stained for PCNA (red nuclei) (J, M) and *survivin *mRNA (white cytoplasm) (K, N) and the confocal images were overlaid (L, O). Only a subpopulation of PCNA+ cells express *survivin*. EGL, external germinal layer; OB, olfactory bulb; LV, lateral ventricle. Scale bars: A, B 1000 μm; C, F, G 500 μm; D 50 μm; E 200 μm; H, I 100 μm, J-O 20 μm.

Postnatally, survivin expression was restricted largely to rapidly proliferating cells [21] and migrating NPCs [27]. At P7, survivin mRNA was detected in the SVZ, the RMS, the OB, and the dentate gyrus (DG) (Figure 2A, C, D, E). Within the SVZ, survivin was not expressed in the ependymal cells immediately adjacent to the lateral ventricle (Figure 2C, D). During the first two postnatal weeks, survivin mRNA was also detected in granule cell precursors in the external germinal layer (EGL) of the developing cerebellum (Figure 2A). In the adult brain, survivin expression remained restricted to proliferating and migrating precursor cells in the SVZ, the RMS, and the subgranular zone (SGZ) of the DG (Figure 2F, H).

In both the SVZ (Figure 2J-L) and the SGZ of the DG (Figure 2M-O), >95% of survivin expressing cells were positive for the proliferation marker PCNA, while at both sites, 25-50% of PCNA positive cells expressed survivin. Thus, survivin expressing cells represent a subpopulation of the mitotically active cells. Doublecortin (DCX), present in immature, migrating neuroblasts [28] also overlapped with survivin in the SVZ, the RMS and the SGZ. However, similar to PCNA, not all DCX positive cells expressed survivin, indicating that survivin is restricted to a subpopulation of cells in the SVZ-RMS (not shown). Mature neuronal marker NeuN [29] immunoreactivity did not overlap with survivin, consistent with the lack of survivin expression by mature neurons (Figure 2C-E).

Overall, survivin expression is restricted during fetal development to NPCs in neurogenic regions of the telencephalon, of which a fraction populates the major neurogenic regions of the postnatal brain, i.e. the SVZ and the SGZ. Within these neurogenic regions, a subpopulation of NPCs continues to express survivin, which is downregulated once the cells differentiate into neurons. In both the embryo or adult, survivin is not detected in neurons in the cortex, OB, or hippocampus.

### *In vivo Prenatal Survivin *gene inactivation

To study the *in vivo *role of NPC survivin, we generated mice in which the *survivin *gene is inactivated in the neurogenic regions of the brain prenatally. Mice expressing cre recombinase driven by the *CamKIIα *promoter [30,31] were bred with mice in which the entire *survivin *gene is flanked by loxP sites [32]. The resultant *CamKIIα-cre:survivin*^*lox/lox *^(referred to as *Survivin*^*Camcre*^) mice were born in the expected Mendelian distribution, i.e. there was no evidence of embryonic lethality.

We confirmed previous reports of cre recombinase activity in the CAMKII*α-cre *embryos and mice [30,31] by breeding the CAMKII*α-cre *mice with the ROSA26 reporter mice, followed by immunohistochemical detection of GFP. Cre recombinase activity at E12.5 was detected prominently in the ventral telencephalon (ganglionic eminences), but less in the dorsal telencephalon, and not in the eye (Additional file 1: Supplemental Figure S1). Postnatally, it was restricted to postmitotic NeuN positive neurons in the hippocampus and cortex, with lower levels in the striatum, thalamus, hypothalamus and amygdala [30]. Postnatal cre expression was absent in NPCs in the SVZ and SGZ (Additional file 1: Supplemental Figure S2). Thus, cross-breeding with the *Survivin*^*lox/lox *^mice resulted in deletion of both *survivin *alleles in the neurogenic regions in the prenatal period (ganglionic eminences and neocortex).

### Neurogenesis defects in *Survivin*^*Camcre *^embryos

**Figure 3:**
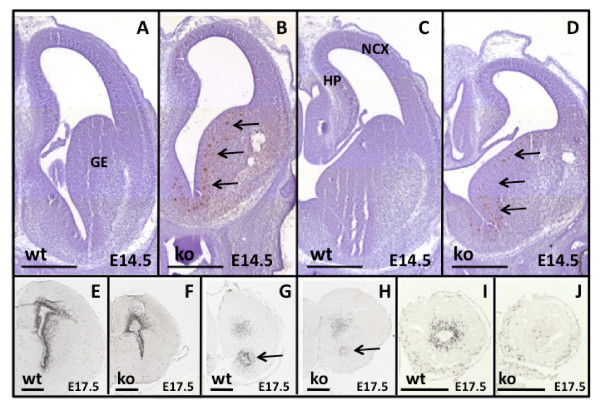
**Neurogenesis defects in *Survivin*^*Camcre *^embryos**. **(A-D) **Tunel labeling of coronal sections of E14.5 brains illustrates increased number of apoptotic cells in the ganglionic eminences (GE) of *Survivin*^*Camcre *^embryos (arrows). **(E-J) **BrdU immunostaining of coronal sections of E17.5 embryos reveals reduced cell proliferation in the SVZ, the RMS (arrow in G, H), and the OB of *Survivin*^*Camcre *^embryos as compared to controls. NCX, neocortex; HP, hippocampus. Scale bars: 500 μm

Cre excision of *survivin *was assessed by *in situ *hybridization in *Survivin*^*Camcre *^and corresponding control *Survivin*^*lox/lox *^embryos. At E14.5 and E17.5, survivin expression in the *Survivin*^*Camcre *^embyros was markedly reduced in the ganglionic eminences surrounding the lateral ventricles where mitotically active NPCs normally reside, in the RMS and in the OB (Figure 1F, H, J, L). This was associated with increased tunel staining, most evident in the ganglionic eminences, and minimally in the dorsal telencephalon (Figure 3A-D). BrdU labeling studies revealed decreased NPC proliferation in the SVZ, RMS and OB of E17.5 *Survivin*^*Camcre *^embryos (Figure 3E-J). Thus, lack of NPC survivin results in increased embryonic NPC death and decreased NPC proliferation in specific embryonic neurogenic regions.

### Altered postnatal neurogenesis in *Survivin*^*Camcre *^mice

**Figure 4:**
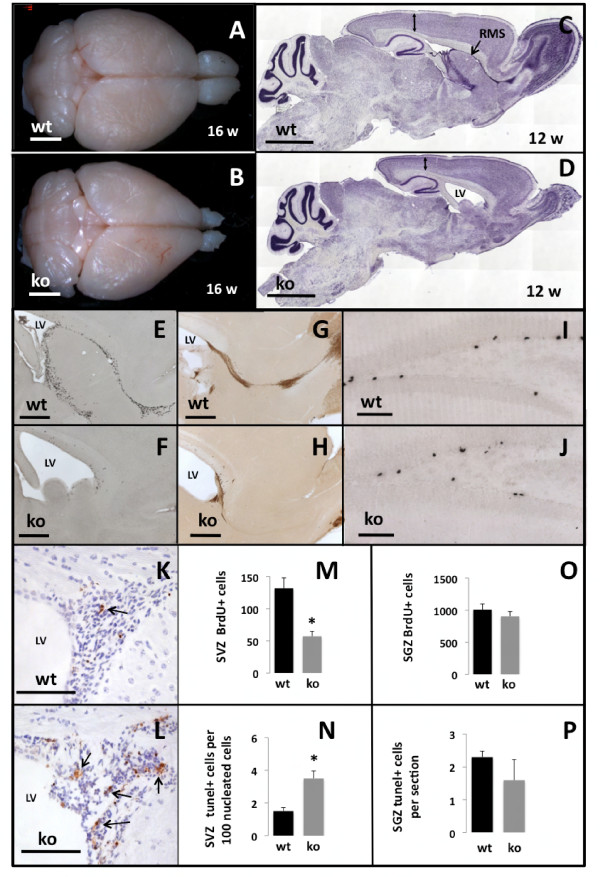
**Altered neurogenesis in adult *Survivin*^*Camcre *^mice**. **(A, B) **Dorsal view of control (wt) and *Survivin*^*Camcre *^(ko) whole brains at 16 weeks, reveals OB hypoplasia in *Survivin*^*Camcre *^mice. **(C, D) **Nissl stained sagittal sections of littermates, shows thinner cortex (double arrow) and absence of the RMS in the *Survivin*^*Camcre *^mice. **(E, F, I, J) **BrdU labeling of sagittal sections of brains from 12 week old mice shows proliferation in the SVZ-RMS pathway (E, F) and in the SGZ (I, J), that is reduced only in the SVZ of *Survivin*^*Camcre *^mice (F) as compared with controls (E). **(G, H) **DCX immunostaining of sagittal sections through the forebrain of control (G) and *Survivin*^*Camcre *^(H) mice. **(M, O) **Quantification of BrdU^+ ^cells in the SVZ (M) and the SGZ (O) was performed as detailed in Methods. There was a significant reduction in BrdU+ cells in the SVZ, but not in the SGZ of *Survivin*^*Camcre *^mice as compared to controls. **(K, L) **Coronal sections through the anterior SVZ of control (K) and *Survivin*^*Camcre *^(L) mice were stained for tunel+ apoptotic cells (arrows). **(N, P) **The number of tunel+ cells in the SVZ (N) and the SGZ (P) of control and *Survivin*^*Camcre *^mice was quantified as described in Methods. There was a significant increase in tunel+ cells in the SVZ but not in the SGZ of *Survivin*^*Camcre *^mice as compared to controls. LV, lateral ventricle. Results in panels M, N, O, P are reflected as means + SEM, n = 4-5 mice per group. *P < 0.005. Scale bars: A-D 2 mm; E-H 500 μm; I-L 100 μm. (C-L) 12 weeks.

Body weights of *Survivin*^*Camcre *^and control mice were not significantly different at birth. However, during the first month after birth, *Survivin*^*Camcre *^mice had a significantly higher mortality rate of 28% (n = 27/95) compared to 0% (n = 0/97) in control mice. Adult *Survivin*^*Camcre *^mice also had significantly smaller brains, and the OBs were strikingly hypoplastic (Figure 4A, B) (for brains: 421 ± 11 gm *versus *329 ± 7 gm, for controls *versus Survivin*^*Camcre*^, respectively, p < 0.001; for OBs: 19.7 ± 0.9 gm *versus *3.5 ± 0.2 gm for controls *versus Survivin*^*Camcre*^, p < 0.001; n = 6 mice per group). *In situ *hybridization confirmed loss of survivin expression in the SVZ and RMS (Figure 2A, B, F, G). Surprisingly, expression of survivin in the SGZ was not decreased postnatally in *Survivin*^*Camcre *^mice (Figure 2I). Nissl stained brain sections from *Survivin*^*Camcre *^mice revealed loss of the RMS, decreased cortical thickness (average reduction to 80% of control at bregma levels -1.34/-1.70/-2.46/-2.80 mm; n = 4 mice per group, p < 0.01 at each bregma level), enlarged ventricles, yet no morphological changes in the hippocampus (Figure 4C, D). The latter was confirmed by quantifying the volumes corresponding to the GCL and the hilus of the DG (GCL: 0.11 ± 0.01 mm^3 ^*versus *0.12 ± 0.01 mm^3 ^for control and *Survivin*^*Camcre *^mice, respectively; hilus: 0.11 ± 0.01 mm^3 ^*versus *0.15 ± 0.02 mm^3 ^for control and *Survivin*^*Camcre *^mice, respectively; n = 4-5 mice per group, p > 0.05).

The hypoplastic OB and absent RMS, in concert with reduced expression of NPC survivin in the *Survivin*^*Camcre *^mice might be caused by decreased NPC proliferation, increased cell death, and/or deficits in migration. Accumulation of cells in the anterior SVZ of the *Survivin*^*Camcre *^mice was not observed, mitigating against a predominant migration defect. NPC proliferation, assessed 1 hr after a single dose of BrdU, revealed a significant reduction in BrdU labeled cells in the SVZ of the *Survivin*^*Camcre *^mice (132 ± 16 cells *versus *57 ± 8 cells for control and *Survivin*^*Camcre *^mice, respectively, n = 4-5 mice per group, p = 0.002) (Figure 4E, F, M). Furthermore, both BrdU and DCX labeled cells were almost completely absent in the RMS, in striking contrast to the controls (Figure 4E-H). Cell death in the SVZ of the *Survivin*^*Camcre *^mice was also significantly increased (Figure 4K, L, N), as quantified by the number of tunel+ cells in the anterior SVZ (1.5 + 0.21/100 nucleated cells *versus *3.5 + 0.46 tunel+ cells/100 nucleated cells, for control and *Survivin*^*Camcre *^mice, respectively, n = 4-5 mice per group, p = 0.004) (Figure 4N). In the RMS and OB, there were some tunel+ cells identified in the brains of the control mice, but not in *Survivin*^*Camcre *^mice, the latter likely due to the lack of precursor cells in this region.

As noted above, survivin expression in the SGZ of the DG was not appreciably diminished after cre excision. There was also no alteration in the number of proliferating or immature neurons in the SGZ, as quantified by BrdU labeling (1010 + 88 *versus *905 + 75 cells in controls and *Survivin*^*Camcre *^mice, respectively, n = 4-5 mice per group, p = 0.39) (Figure 4I, J, O) or DCX immunoreactivity (not shown). Nor could we detect changes in tunel+ staining (2.3 + 0.18 *versus *1.6 + 0.63 tunel+ cells/section in controls and *Survivin*^*Camcre *^mice, respectively, n = 3-4 mice per group, p = 0.30) (Figure 4P).

In summary, striking survivin-dependent defects in neurogenesis are evident postnatally in the RMS and OB of *Survivin*^*Camcre *^mice, due to a combination of increased SVZ NPC apoptosis and diminished cellular proliferation. Despite the fetal abnormalities, and in striking contrast to the RMS-OB, there were no obvious structural defects or alterations in hippocampal neurogenesis in the *Survivin*^*Camcre *^mice that had no appreciable reduction in survivin expression within the hippocampus.

### Embryonic survivin administration increases neurogenesis

In pilot studies, we assessed whether prenatal administration of survivin to increase expression in NPCs could promote neurogenesis. E12.5 control *Survivin*^*lox/lox *^and *Survivin*^*Camcre *^embryos received an intracerebroventricular injection *in utero *with the lentiviral vector pCHMWS-eGFP-T2A-SRV140 (survivin vector) or pCHMWS-eGFP-T2A-Fluc (control vector). At P21, immunohistochemical analysis of the control *Survivin*^*lox/lox *^mice that received the survivin vector revealed an increased number of embryonic precursor cell-derived cells in the OB as compared with the control vector (Additional file 1: Supplemental Figures S3A, B). With *Survivin*^*Camcre *^embryos, the survivin vector did not apparently reverse the OB hypoplasia when examined at P21, but there were notably more embryonic precursor cell-derived cells in the OB of 3 out of 3 *Survivin*^*Camcre *^mice that received the survivin vector, as compared with the 2 *Survivin*^*Camcre *^mice that received the control vector (Additional file 1: Supplemental Figures S3C, D). Overall, these preliminary findings suggest that enhanced expression of NPC survivin may increase neurogenesis.

### *Survivin*^*Camcre *^mice have a thinner cortex with fewer GABAergic interneurons and a lower seizure threshold

**Figure 5:**
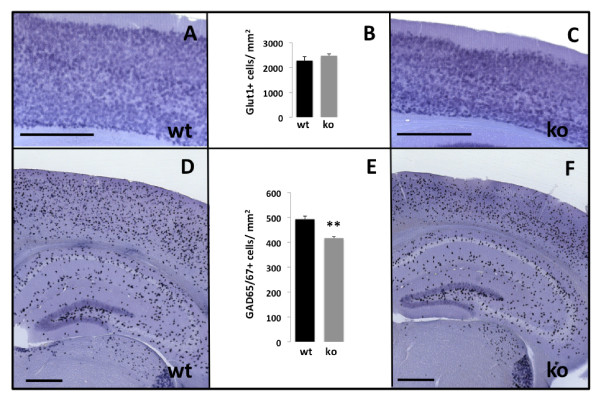
**GABAergic and glutamatergic inter/neurons**. *In situ *hybridizations of coronal sections were performed to detect and quantify vGLUT1+ glutamatergic neurons (A-C) and GAD65/67+ GABAergic interneurons (D-F) in adult littermates. **(A-C) **In spite of *Survivin*^*Camcre *^mice having a thinner cortex, the density of vGLUT1+ cells in the cortex was not signficantly different between *Survivin*^*Camcre *^and control mice. **(D-F) **The density of GAD65/67+ cells in the cortex was significantly reduced in *Survivin*^*Camcre *^mice, but not in the hippocampus (see text). Results in panels B and E are reflected as means + SEM, n = 4-6 mice per group. **P < 0.001 Scale bars: 500 μm.

Although the average cortical thickness in the *Survivin*^*Camcre *^mice was reduced, *in situ *hybridization with cortical markers (Cux2 for layers 2-4 [33], Badlamp for layers 2/3/5 [34], and ER81 for layer 5 [35] confirmed the correct orientation and presence of all the cortical layers (not shown). Moreover, and in line with limited expression of cre recombinase in the dorsal telencephalon of *CamKIIα-cre *embryos, the density of cortical vGLUT1+ glutamatergic cells in the *Survivin*^*Camcre *^mice was not altered (2282 + 157 cells/mm^2 ^*versus *2480 + 71 cells/mm^2 ^in controls and *Survivin*^*Camcre *^mice, respectivley, n = 4-5 mice per group, p = 0.30) (Figure 5A-C). In contrast, and consistent with prominent apoptosis and diminished BrdU labeling in the ganglionic eminences, prenatal depletion of survivin in the NPCs of *Survivin*^*Camcre *^mice resulted in a significant reduction in the density of GAD65/67+ GABAergic interneurons in the postnatal adult cortex (493 + 12 cells/mm^2 ^*versus *417 + 6 cells/mm^2 ^in controls and *Survivin*^*Camcre *^mice, respectively, n = 5-6 mice per group, p < 0.001) (Figure 5D-F). This occurred in the absence of any changes in interneurons in the hippocampus (hilus + granular cell layer: 213 ± 8 cells/mm^2 ^*versus *209 ± 17 cells/mm^2 ^in controls and *Survivin*^*Camcre *^mice, respectively, n = 4 mice per group, p = 0.82). These data indicate an alteration in the excitatory/inhibitory balance in the brain of *Survivin*^*Camcre *^mice that might be associated with postnatal alterations in cognition and behavior, and a lower seizure threshold.

**Figure 6:**
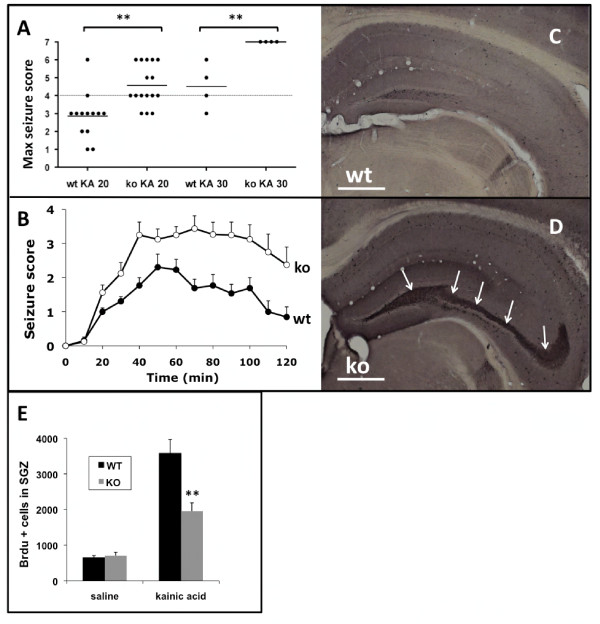
***Survivin*^*Camcre *^mice exhibit increased seizure activity**. **(A) **Scatter plot showing the maximum seizure score assigned to each experimental animal during a 2 hr observation period following KA administration. Seizure scores were significantly higher in the *Survivin*^*Camcre *^mice as compared to controls. Horizontal bars indicate the mean for each group. **(B) **KA (20 mg/kg ip) induced signficantly more severe seizure activity in *Survivin*^*Camcre *^mice as compared to controls, P < 0.001, n = 13-16 mice per group. **(C, D) **Representative NPY-stained coronal sections through the hippocampus of saline-treated control (wt) (C) and *Survivin*^*Camcre *^mice (ko) (D) reveals ectopic NPY expression by mossy fibers in *Survivin*^*Camcre *^mice (arrows in D). **(E) **Quantification of BrdU^+ ^cells (1 day after BrdU injection) in the SGZ from saline and KA treated control and *Survivin*^*Camcre *^mice. The neurogenic response to KA was significantly dampened in *Survivin*^*Camcre *^mice as compared to controls, n = 4-5 mice per group. Results in panels B and E are reflected as means + SEM. **P < 0.001. Scale bars: C-D 500 μm.

Indeed, during routine handling, 2.5% (n = 9/352) of the *Survivin*^*Camcre *^mice were recorded to have spontaneous tonic-clonic, generalized motor seizures starting from 2 weeks of age (no seizures observed in controls). To investigate seizure susceptibility, the response of control and *Survivin*^*Camcre *^mice to kainic acid (KA) was assessed over a period of 2 hours. Control, saline-treated animals (n = 9 per group) showed no signs of epileptic activity. However, in response to KA, *Survivin*^*Camcre *^mice exhibited a lower threshold for seizures that were more severe. Thus, at a subconvulsive dose of 20 mg/kg, KA induced limbic motor convulsions in 15% (n = 2/13) of control mice and 81% (n = 13/16) *Survivin*^*Camcre *^mice (Figure 6A). The maximum seizure score was significantly higher in the *Survivin*^*Camcre *^mice (p < 0.001). Seizure severity over the 2 hr observation period was also significantly greater in the *Survivin*^*Camcre *^mice (p < 0.001) (Figure 6B). At a KA dose of 30 mg/kg, the mean latency to the first seizure was also shorter in the *Survivin*^*Camcre *^mice compared to control mice (8.75 ± 2.75 *versus *30 ± 7.64 min, respectively, n = 4 mice per group, p = 0.031). At that dose, all *Survivin*^*Camcre *^mice rapidly developed status epilepticus (stage 5-6) and died of severe generalized convulsions, while all control animals survived the KA treatment (Figure 6A). Thus, *Survivin*^*Camcre *^mice exhibit enhanced susceptibility to KA seizures.

Neuropeptide Y (NPY) is a multifunctional peptide that is expressed in GABAergic interneurons, regulates pre-synaptic excitatory transmission in the DG, and has anti-epileptic properties [36]. Hilar NPY interneuron degeneration, ectopic expression of NPY in mossy fibers, and axonal sprouting are common features of limbic hyperexcitability [37]. Due to the increased seizure activity in the *Survivin*^*Camcre *^mice, we examined NPY expression under both basal conditions (saline treatment) and following induction of seizures (KA 20 mg/kg i.p.). The number of hilar NPY+ interneurons was not different between saline-treated control and *Survivin*^*Camcre *^mice (490 ± 17 *versus *398 ± 53 cells/mm^2^, for control and *Survivin*^*Camcre *^mice, respectively; n = 4-5 mice per group, p = 0.11). However, two weeks after 20 mg/kg KA treatment, there were significantly fewer NPY+ cells in the hilus of the *Survivin*^*Camcre *^mice (446 ± 22 *versus *306 ± 65 cells/mm^2 ^for control and *Survivin*^*Camcre *^mice, respectively; n = 6-9 mice per group, p = 0.032). Moreover, ectopic NPY expression in mossy fibers was readily detected in 3 out of 4 saline-treated *Survivin*^*Camcre *^mice but not in any of the corresponding controls (n = 5) (Figure 6C, D). This effect became more prominent after KA (5/6 *Survivin*^*Camcre *^mice as compared to 0/9 controls). In 2 of these *Survivin*^*Camcre *^mice, we furthermore observed ectopic NPY immunoreactivity in the supragranular layer, likely reflecting sprouting of mossy fibers (not shown).

Since seizure activity modulates hippocampal neurogenesis, we also evaluated the seizure-induced neurogenic response of the control and *Survivin*^*Camcre *^mice. The volumes of the GCL and the hilus were not different between control and *Survivin*^*Camcre *^mice (see above), and KA had no effect on that relationship (data not shown). To assess cell proliferation, BrdU was injected 3 days after KA or saline injection, and mice were sacrificed 1 day later. After saline injection, the total number of SGZ BrdU+ cells was not different in control and *Survivin*^*Camcre *^mice (648 ± 58 cells *versus *705 ± 99 cells in controls and *Survivin*^*Camcre *^mice, respectively, n = 4 mice per group, p = 0.64) (Figure 6E). Compared to saline treated controls, KA treated mice exhibited an increase in the number of BrdU+ cells in the SGZ after KA injection. However, the neurogenic response was significantly dampened in the *Survivin*^*Camcre *^KA treated mice compared to control KA treated mice (3578 ± 392 cells *versus *1955 ± 233 cells for controls and *Survivin*^*Camcre *^mice, respectively, n = 5 mice per group, p < 0.001) (Figure 6E). Numbers of BrdU+ cells remained reduced 2 weeks following KA in the *Survivin*^*Camcre *^mice as compared to controls (data not shown). Thus, despite the higher seizure scores following KA in the *Survivin*^*Camcre *^mice, there was less seizure-induced neurogenesis in the *Survivin*^*Camcre *^*versus *the control mice.

### *Survivin*^*Camcre *^mice exhibit learning and memory defects

Adult *Survivin*^*Camcre *^mice exhibited several defects that may contribute to disorders in behavior and cognition, including reduced SVZ-RMS-OB neurogenesis [38,39], OB hypoplasia [40], diminution of cortical GABAergic neurons, and seizures. We therefore evaluated the effects of depleting NPCs of survivin by subjecting the *Survivin*^*Camcre *^and matched controls to a range of behavioral studies.

There was no difference in body weight between the *Survivin*^*Camcre *^and control mice at the start of behavioral testing (21.6 ± 0.6 gm *versus *20.1 ± 0.6 gm for the *Survivin*^*Camcre *^and controls, respectively, p = 0.081), and the *Survivin*^*Camcre *^mice had normal visual and auditory skills, grip strength, rotarod performance, pain response and cage activity (Additional file 1: Supplemental Figures S4, S5; Additional file 2: Supplemental Methods and Results).

**Figure 7:**
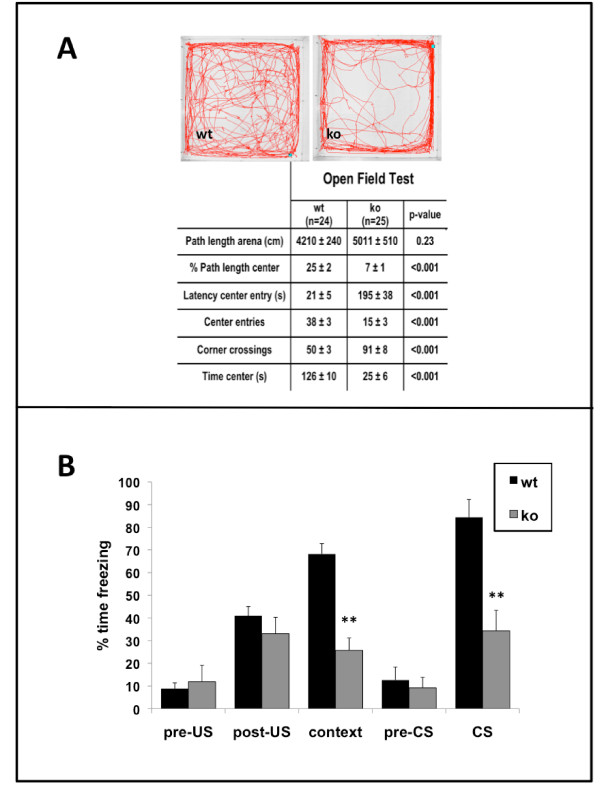
**Open field and fear conditioning defects in *Survivin*^*Camcre *^mice**. **(A) **Open field test data are provided, with representative paths of control (wt) and *Survivin*^*Camcre *^(ko) mice. **(B) **Contextual and auditory-cued fear conditioning in control and *Survivin*^*Camcre *^mice. Freezing times in pre-US, post-US and pre-CS trials were not different between control and *Survivin*^*Camcre *^mice. *Survivin*^*Camcre *^mice showed significantly less freezing responses as compared to controls during both the context and the auditory cue (CS) trials. US: unconditioned stimulus, shock; CS: conditioned stimulus, auditory cue. Results in panel B are reflected as means + SEM, n = 12 mice per group. **P < 0.001.

In the open field test, the *Survivin*^*Camcre *^mice displayed significant disturbances in exploratory behavior, including delayed first entry to the center, fewer center entries, more corner crossings, and less time in the center (Figure 7A). This type of behavioral outcome is often suggestive of increased anxiety, however the *Survivin*^*Camcre *^mice performed normally in the elevated plus maze test for anxiety. On the maze, there was no difference between control and *Survivin*^*Camcre *^mice in the total number of beam crossings (148 ± 5 *versus *155 ± 9 for control and *Survivin*^*Camcre *^mice respectively; n = 12 mice per group, p = 0.48), percent time spent in the open arms (33 ± 2 *versus *36 ± 4 for control and *Survivin*^*Camcre *^mice respectively; n = 12, p = 0.76), nor percentage of entries into the open arms (26 ± 2 *versus *23 ± 3 for control and *Survivin*^*Camcre *^mice respectively; n = 12, p = 0.07). The findings suggest that the poor performance of the *Survivin*^*Camcre *^mice in the open field test may not be due to increased anxiety, but rather due to a distinct defect in exploratory behavior.

The *Survivin*^*Camcre *^mice exhibited a significant impairment in passive avoidance learning. During the initial training trial, there was no difference in step-through latencies (12.5 ± 4.5 *versus *13.9 ± 1.8 sec, *Survivin*^*Camcre *^and control mice respectively, p = 0.783). However, during testing, the *Survivin*^*Camcre *^mice demonstrated significantly shorter latency to enter the dark compartment than the controls (235 ± 27 *versus *71 ± 24 sec for control and *Survivin*^*Camcre *^mice, n = 12, p < 0.001) (Additional file 1: Supplemental Figure S6), consistent with poor associative memory of aversive stimuli.

We examined auditory and contextual fear memory using an auditory cue as the conditioned stimulus (CS), and a foot shock as an aversive stimulus. Freezing times in baseline, pre-US, post-US, and pre-CS trials were not different between the groups (Figure 7B). However, *Survivin*^*Camcre *^mice exhibited significantly less freezing response in the context and auditory cue (CS) trials compared to control mice (context: 25.7 ± 5.5 *versus *68.2 ± 4.5; auditory: 34.3 ± 9.0 and 84.4 ± 7.8, respectively for *Survivin*^*Camcre *^and control mice, n = 12 mice per group, p < 0.001) (Figure 7B).

**Figure 8:**
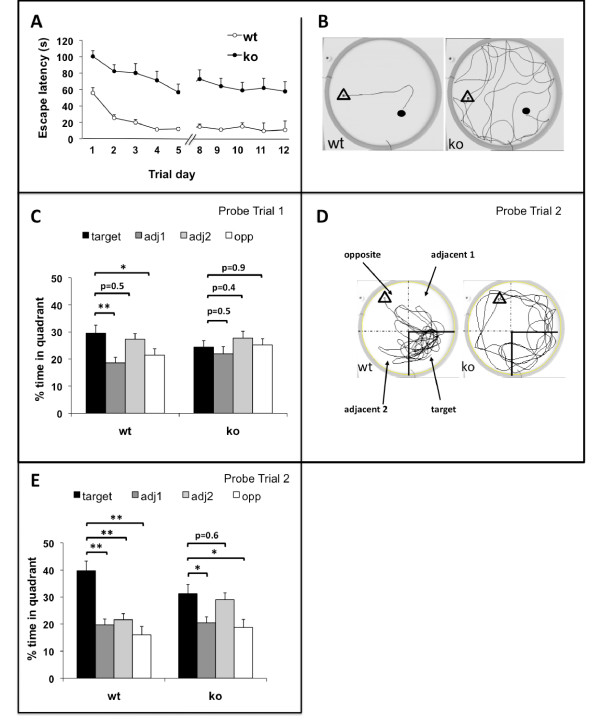
**Poor performance of *Survivin*^*Camcre *^mice in water maze**. Morris water maze studies were performed as detailed in Methods. **(A) ***Survivin*^*Camcre *^mice (ko) exhibited a significantly longer escape latency as compared to controls (wt) during acquisition of the task (P < 0.001). **(B) **Representative swim paths of control and *Survivin*^*Camcre *^mice during acquisition training. **(C) **The mean percent time spent in each quadrant during the 1st probe trial is plotted for both genotypes. In the first probe trial performed after 5 days of training, the control mice already had a preference for the target quadrant compared to adjacent 1 and opposite quadrants, whereas the *Survivin*^*Camcre *^mice had no preference at all. **(D, E) **In the second probe trial (representative swim path shown in D), control mice spent most of the time in the target quadrant, while *Survivin*^*Camcre *^mice spent equal amounts of time in the target and adjacent 2 quadrant. Open triangle and black dot represent location of the start and the platform, respectively. adj, adjacent 1 or adjacent 2 quadrant; opp, opposite quadrant. Results in panels A, C and E are reflected as means + SEM, n = 12 mice per group. *P < 0.05; **P < 0.001.

Lastly, we assessed hippocampus-dependent spatial learning and long-term memory in the *Survivin*^*Camcre *^mice using the Morris water maze. Swimming velocity was not different between the controls and the *Survivin*^*Camcre *^mice, excluding defects in motor ability. During acquisition training, the *Survivin*^*Camcre *^mice showed training-dependent reduction in escape latency and path length. However, the improvements were minimal as compared with controls, and the escape latency and path length were significantly increased in the *Survivin*^*Camcre *^mice (p < 0.001, n = 12; by two way repeated measures ANOVA) (Figure 8A, B). Notably, lengths of the escape paths were not different between the *Survivin*^*Camcre *^mice and control mice during 4 visible-platform training days (803 + 88 cm *versus *1076 + 110 cm for control, n = 10, and *Survivin*^*Camcre *^mice, n = 12, respectively; p = 0.075). These results indicate that the *Survivin*^*Camcre *^mice were fully capable and motivated to choose the shortest pathway towards the platform in the cued, non-spatial condition of the task.

In the first probe trial performed after 5 days of training, the control mice already had a preference for the target quadrant compared to adjacent 1 (p = 0.002) and opposite (p = 0.02) quadrants, whereas the *Survivin*^*Camcre *^mice had no preference at all (p = 0.46) (Figure 8C). In the second probe trial after 10 days of training, the control mice continued to show a strong preference for the target quadrant compared to all other quadrants (p < 0.001), whereas the *Survivin*^*Camcre *^mice equally favoured the target and adjacent 2 quadrants (p = 0.60) *versus *the other 2 quadrants (p < 0.05) (Figure 8D, E). Since the Morris water maze test is a stress that might alter neural cell proliferation, we also quantified the number of Ki67+ cells in the dentate gyrus of the *Survivin*^*Camcre *^mice (n = 4) and the corresponding littermate controls (n = 4) after the probe trials. There was no significant difference in the number of Ki67+ cells between the two groups (534 + 49 cells *versus *480 + 28 cells in controls *versus Survivin*^*Camcre *^mice, respectively, p = 0.378).

Overall, the *Survivin*^*Camcre *^mice exhibited exploratory behavioral abnormalities, with global deficits in various forms of learning and memory.

## Discussion

In this report, we show that survivin is prominently expressed in the neurogenic regions of the embryonic mouse brain, and that its expression by a subpopulation of NPCs is maintained postnatally in the two key sites of adult neurogenesis - the SVZ and the SGZ. Lack of expression of survivin in the NPCs during embryonic development, was associated with profound SVZ-RMS-OB postnatal defects in neurogenesis and loss of interneurons, manifest by major deficits in learning and memory, and heightened sensitivity to seizures. Prenatal administration of survivin in the brain enhanced neurogenesis in the SVZ-RMS-olfactory system. Our findings position survivin as a central player in regulating neurogenesis during embryonic development, alterations of which impact on postnatal brain function.

The embryonic forebrain, the telencephalon, consists of two parts. The dorsal aspect is the origin of glutamatergic excitatory neurons of the cerebral cortex and hippocampus [41]. The ventral part, comprising the ganglionic eminences, gives rise to the basal ganglia. The LGE provides neurons for the striatum [42], interneurons of the olfactory bulb (OB) [43], and most adult SVZ NPCs [2]. The MGE is the source of most neocortical [42] and hippocampal interneurons [44], as well as striatal interneurons. At E12.5, survivin is widely expressed in the neurogenic region of the ventral and dorsal telencephalon. Since cre recombinase expression in the *CamKIIα-cre *mice is low in the dorsal telencephalon, generation of principal glutamatergic neurons was largely unaffected in the adult, and the overall integrity of the hippocampus and cortex was maintained, albeit the latter was thinner. In contrast, the number of cortical GABAergic neurons, which arise primarily in the ganglionic eminences and comprise 25-30% of cortical neurons, was significantly reduced in the *Survivin*^*Camcre *^mice. This reduction may have been further contributed to by the paucity of SVZ NPCs, recently shown to be a continuous postnatal source of GABAergic interneurons in the cortex [45].

Imbalances in inhibitory and excitatory circuits due to decreases in numbers of interneurons, are well known to be associated with seizures in humans and experimental animal models [46,47]. This was clearly evident in the *Survivin*^*Camcre *^mice which, even under naïve conditions, displayed spontaneous, generalized tonic-clonic motor seizures, a phenotype that was more dramatically revealed following challenge with KA. Indeed, *Survivin*^*Camcre *^mice showed a rapid and consistent generalization of seizures at KA doses that normally result in focal hippocampal epileptic activity [48]. Thus, a defect in the cortical inhibitory system may explain the higher susceptibility to generalized convulsions in the *Survivin*^*Camcre *^mice.

Our studies demonstrate that the loss of a subpopulation of NPCs in the SVZ of neonatal and adult *Survivin*^*Camcre *^mice, with resultant near-absence of the RMS and OB, was due to a combination of increased apoptosis and decreased cellular proliferation of NPCs in the corresponding embryonic neurogenic region (ganglionic eminences). Indeed, this is in line with the fact that survivin is a pro-survival molecule with the capacity to inhibit apoptosis and to promote the cell cycle and mitosis (reviewed in [49]). Somewhat surprisingly, in spite of profound disturbances in neurogenesis in the SVZ, we did not detect baseline changes in neurogenesis in the SGZ of the DG in the *Survivin*^*Camcre *^mice, or significant loss of survivin expressing DG NPCs. Although this may be due to cre recombinase inefficiency, the finding may also be due to the embryonic origin of SGZ NPCs being different from the SVZ NPCs, which still remains to be clarified [50]. There is however, a defect in SGZ neurogenesis in the *Survivin*^*Camcre *^mice that is only evident under stress conditions. This may mean that the baseline source(s) of SGZ NPCs is different from that recruited during stress, an hypothesis that requires testing. In the *Survivin*^*Camcre *^mice, the neurogenic response was significantly impaired as compared to the controls after KA-induced seizures. Alterations in GABA signaling in the *Survivin*^*Camcre *^mice may be implicated [51], but other factors that are important in maintaining the function of the neurogenic niche in the hippocampus could also contribute to the dampened response [52]. Further study to identify those that are relevant is ongoing.

Although the integrity of the hippocampus was apparently maintained under baseline conditions, upon testing, the *Survivin*^*Camcre *^mice exhibited striking defects in memory and cognition, that are consistent with hippocampal dysfunction. In fact, the behavioral abnormalities were associated with a significant impairment of long-term potentiation (LTP) in the CA1 region of the hippocampus (not shown), a finding that frequently is associated with poor memory, and often with increased epileptic activity. As with the seizure disorder, a loss of cortical inhibitory interneurons likely contributed to the behavioral abnormalities and cognitive defects in the *Survivin*^*Camcre *^mice. We also cannot exclude a contribution of suboptimal neurogenic responses to the behavioral phenotype, as neurogenic defects in both the SGZ and SVZ have been implicated in memory, cognition, mood, and hippocampal-dependent learning [53]. Moreover, olfactory bulbectomy in rodents impairs neurogenesis in both the SGZ and the SVZ, disrupts normal hippocampal LTP, and causes significant deficits in learning and memory. Thus, the OB, which sends projections to the hippocampus [54], also plays a role in normal behavior and cognition [12,13,40]. Indeed, since the *Survivin*^*Camcre *^mice have major defects in neurogenesis, as well as notable hypoplasia of the OB, all of which are associated with epileptic activity and major alterations in behavior, it is reasonable to consider that the effective lack of an OB exacerbates the loss of SVZ and possibly SGZ NPCs, which in turn, contributes to the behavioral abnormalities and enhanced seizure activity.

## Conclusions

We have established that prenatal expression of survivin in neurogenic regions of the developing brain plays a key role in learning and memory and in determining seizure susceptibility. Prenatal stresses are recognized to suppress postnatal neurogenesis that in turn, induces behavioral abnormalities in the neonate and adult [8,9]. While the underlying molecular mechanisms have not been delineated, it is reasonable to consider that alterations in embryonic NPC survivin expression might contribute to those phenotypic changes. Pilot data indicate that prenatal administration of survivin can enhance neurogenesis in the olfactory system. We do not yet know whether the resultant new neurons differentiate or integrate, or whether the SGZ is also affected. Nonetheless, the findings are promising, supporting the critical nature of this molecule, and its potential as a therapeutic target. Our mouse model provides the opportunity to elucidate the relevance of survivin-expressing NPC subpopulations *in vivo *in response to a range of environmental stresses, and genetic or epigenetic factors.

## Methods

### Transgenic mice and genotyping

Mice that express Cre recombinase driven by the promoter of the gene for calmodulin-dependent protein kinase IIα (CamKIIα) [30,31] (gift of Dr. G. Schütz, Heidelberg, Germany) were bred with mice in which the *survivin *gene is flanked by loxP sites [32]. The resulting offspring that were heterozygous for Cre and homozygous for floxed *survivin *(*Survivin*^*lox/lox*^) (hereafter referred to as *Survivin*^*Camcre *^mice) were compared to littermate control mice which did not express Cre and were *Survivin*^*lox/lox*^. *Survivin*^*lox/lox *^embryos and adults were not different from *Survivin*^*lox/wt*^, *Survivin*^*wt/wt *^or *CamKIIα-cre:survivin*^*lox/wt *^mice. Mice were maintained on a C57B/6:Swiss:129svj 75:12.5:12.5 background. Genotyping of tail DNA was performed by PCR as previously reported [30,32]. Mice were group-housed in standard mouse cages in a room with a 12 h light-dark cycle and *ad libitum *access to food and water and all animal experiments were approved by the ethics committee of the University of Leuven.

### BrdU labeling and quantification

Adult mice and pregnant females were injected intraperitoneally (ip) with 5-bromo-2-deoxyuridine (BrdU, Sigma Aldrich, Bornem, Belgium) at a concentration of 50 mg per kg body weight. For the analysis of the embryos, 1 hour after injection of BrdU, pregnant females were killed by cervical dislocation, after which the embryos were harvested, placed in ice-cold PBS, and then fixed in 4% paraformaldehyde (Para) for cutting 20 μm cryo sections using a microtome/cryostat (HM550, Microm, Walldorf, Germany). For analysis of adults, mice were anesthetized with sodium pentobarbital at 1 hour (unless stated otherwise) after BrdU and perfused transcardially with 0.9% NaCl, followed by fixation with 4% paraformaldehyde. Brains were dissected and post-fixed overnight at 4°C and 40 μm tissue sections were prepared using a vibrating microtome (HM650V, Microm, Walldorf, Germany).

The number of BrdU+ cells in the adult dentate gyrus (DG) was quantified using a modified version of the optical fractionator method [55] with Stereo Investigator software (MicroBrightField, Colchester, VT, USA). Cells were counted with a 40× objective on every sixth section through the entire rostrocaudal extension of one half of the DG, restricted to the subgranular zone (SGZ) [56]. The number of BrdU+ cells in the SVZ of one lateral ventricle was counted with a 40× objective on 1 coronal section (bregma level + 0.14 mm) per animal.

### Immunohistochemistry

Immunostaining protocols were optimized for the different tissue preparations and antibodies. In general, tissue sections were treated with 1% H_2_0_2 _in PBS/methanol for 15 min, incubated in 5% serum for 30 min, and incubated overnight at 4°C in following primary antibodies: rabbit anti-neuropeptide Y (NPY) antibody (1:5000, Bachem, UK); mouse anti-NeuN (1:500, Chemicon, Hofheim, Germany); mouse anti-PCNA (1:1000, Chemicon, Hofheim, Germany); rabbit anti-DCX (1:500, Cell Signaling, MA, USA); rat anti-BrdU (1:500, Immunologicals Direct, Oxford, UK); chicken anti-GFP (1:3000, Aves, Oregon, USA); and rabbit anti-Cre recombinase (1:3000, gift from Dr. Schütz, Heidelberg, Germany); rabbit anti-Ki67 (1:1000 Monosan, Uden, The Netherlands). After washes, the corresponding biotinylated secondary antibody was added for 1 hour and the signal was amplified using the Vectastain Elite ABC kit (Vector Laboratories, CA, USA). Peroxidase activity was detected with 3,3'-diaminobenzidine (DAB peroxidase substrate tablet set, Sigma Aldrich, Bornem, Belgium). For fluorescent staining, Alexa-conjugated secondary antibodies (Molecular Probes, Leiden, The Netherlands) were used. BrdU staining was performed as reported previously [57].

### *In situ *hybridization

Digoxygenin (DIG)-labeled RNA probes for Dlx1 [58], Dlx5 [24], Ngn2 (gift from Dr. A. Simeone), GAD65 [59], GAD67AE [60] and vGLUT1 (Allen Institute for Brain Science) were generated using the DIG RNA Labeling Kit (Roche Diagnostics, Basel, Switzerland), according to the manufacturer's instructions. GAD65 and GAD67AE probes were mixed to detect the total number of GABAergic interneurons. For survivin riboprobes, full-length murine survivin cDNA was cloned into the pcDNA3 plasmid vector (Invitrogen, CA, USA) [61], and linearized for generation of antisense and sense probes using Sp6 RNA polymerase or T7 polymerase, respectively. *In situ *hybridization and combined immunohistochemistry protocols were adapted from those reported [33,62] and completed on 20 μm cryostat or 40 μm vibratome sections.

### Measurement of granular cell layer (GCL) volume and hilar volume

Coronal sections through the DG were stained with cresyl violet. Pictures were taken at 4× magnification, and the area of the GCL and the hilus was determined off line using Metamorph software (Molecular Devices, Sunnyvale, CA). Volumes were calculated and expressed in mm^3^.

### Quantification of apoptosis (tunel+ cells)

Detection of cellular apoptosis in 10 μm coronal paraffin sections, prepared using a HM360 microtome (Microm, Walldorf, Germany), was accomplished using the ApopTag Peroxidase In Situ Apoptosis Detection Kit (Chemicon, Hofheim, Germany). The number of tunel+ cells was counted with a 40× objective. The anterior subventricular zone (SVZa) was analyzed at level bregma +0.98 mm and the data are presented as the number of tunel+ cells per 100 nucleated cells. The subgranular zone (SGZ) was analyzed at bregma levels -1.34/-1.70/-2.46/-2.80 mm and the data are presented as the number of tunel+ cells per section.

### Quantification of inhibitory and excitatory neurons

Numbers of neurons and interneurons were quantified hemilaterally on coronal vibratome sections at 4 bregma levels (-1.34/-1.70/-2.46/-2.8 mm). GAD65/67+ cells were counted with a 10× objective in the hilus plus the granule cell layer, and in the parieto/temporal cortex, in a 1.4 mm wide band from the white matter to the pial surface. vGLUT1+ cells in the parieto/temporal cortex were counted with a 20× objective in a 0.7 mm wide band. NPY+ cells in the hilus were counted with a 20× objective on every sixth section (40 μm thick). Results are presented as the number of cells per mm^2^.

### Quantification of Ki67 positive cells

Ki67 positive cells in the SGZ were counted hemilaterally with a 40× objective on every third 40 μm section between bregma levels -1.34 and -2.8 mm. The number of counted cells was multiplied by 3 to obtain the total number of Ki67 positive cells.

### Intracerebroventricular (ICV) injection of lentiviral vector in embryos

Lentiviral vectors were prepared, encoding enhanced green fluorescent protein (eGFP) and survivin separated by a T2A sequence starting from pCHMWS-eGFP-T2A-Fluc (gift from Dr. V. Baekelandt, KULeuven). The Fluc fragment was removed from pCHMWS-eGFP-T2A-Fluc using *Bam*HI and *Mlu*I and replaced by the cDNA encoding full-length murine survivin. Survivin expression from this vector was confirmed by Western blot analysis of lysates from transfected COS cells. Human immunodeficiency virus type 1 (HIV-1)-derived lentiviral vectors were produced by a standard protocol. The viral vector was mixed with Fast Green dye (0.005% final concentration, Sigma-Aldrich, Bornem, Belgium), which allowed visualization of the distribution of the viral vector in the cerebral ventricles after injection. Pregnant mice (stage E12.5) were anesthetized with 50 mg/ml ketamine, 2% xylazine in saline and placed supine on a heating pad. A 2-cm midline incision was made through the skin and the abdominal wall. The uterine horn was drawn out through the hole onto gauze, and with the uterus transilluminated, a 35 gauge needle (beveled NanoFil needle, World Precision Instruments, FL, USA) was inserted into the ventricle, and 1 μl viral vector solution was injected at a speed of 406 nanoliters per second using a Mycro4® MicroSyringe Pump Controller (World Precision Instruments, FL, USA).

### Seizure studies

Seizures in adult male mice were evoked by ip administration of kainic acid (KA) (Sigma, MO, USA). KA was dissolved in saline and injected at 20 or 30 mg/kg body weight. Saline-injected animals were used as controls. Seizure severity was quantified by an observer blind to the mouse genotype using the following scale [48,63]: stage 0, normal behavior; stage 1, immobility; stage 2, forelimb and/or tail extension, rigid posture; stage 3, repetitive movements, head bobbing; stage 4, rearing and falling; stage 5, continuous rearing and falling; stage 6, severe whole-body convulsions; and stage 7, death. For each animal, seizure severity was scored every 10 min over a period of 2 hours after KA administration. The maximum score reached by each animal over the entire observation period was used to calculate the maximum seizure score for each treatment group. Seizure severity over the 2 hour observation period was calculated for each mouse as the area under the seizure score versus time curve (AUC), and the average AUC was calculated for each treatment group.

### Behavioral studies

Behavioral tests were initiated when the mice were 3-4 months of age, n = 12-25 per group. Neuromotor, exploration, and learning tests were performed in the following sequence: cage activity, grip strength, rotarod, open field, elevated plus maze, Morris water maze, passive avoidance. Contextual fear conditioning was performed on a separate group of mice. Animals were tested during the light phase of the light-dark cycle. All studies were performed by observers who were blinded to the genotype of the mice.

Open field exploratory activity was assessed in a 50 cm × 50 cm arena using EthoVision video tracking and software (Noldus, Wageningen, The Netherlands). Mice were individually placed in a specific corner of the open field, and were allowed a 1 min adaptation period. The path was recorded for 10 min to measure dwells and entries in different parts of the field. Measures included total path length, percentage path length in the center circle (diameter 30 cm), entries into the four corner squares, entries into the center, time spent in the center *versus *periphery, latency of first center approach, and frequency of rearing.

The elevated plus maze [64,65], to evaluate anxiety-like behavior, had two open arms (21 cm × 5 cm) and two closed arms of the same size, with high side walls, and was raised 30 cm above the table. Each mouse was placed in the central square of the maze, facing one of the closed arms. After 1 min, exploratory behavior was recorded automatically during a 10 min period using five infrared beams, connected to an activity logger. For each mouse, the number of arm entries, percentage of open arm entries, and percentage time spent in the open arms was assessed.

Passive avoidance (aversive) learning [66] was tested in a two-compartment step-through box. Animals were adapted to the dark for 30 min, and then placed into a small illuminated compartment. After 5 s, a sliding door leading to the large dark compartment was opened. Upon entry, the door was closed and the animal received an electric foot shock (0.3 mA, 1s). Twenty-four hours later, the animals were placed again in the light compartment and the latency to enter the dark compartment was measured up to 300 s, to evaluate memory of the foot shock.

Contextual and auditory-cued fear conditioning [67,68] was tested in a Plexiglas chamber with a grid floor through which a foot shock could be administered. Mice were trained and tested on 3 consecutive days: On the day 1, the mice were individually placed in the testing chamber and allowed to adapt for 5 min. On the day 2, the animals were allowed to explore the testing chamber for 2 min, after which an auditory cue (conditioned stimulus, CS) was presented for 28 s, followed by a foot shock (0.3 mA, 2 s; unconditioned stimulus, US). The time (%) spent freezing during the first 2 min and 28 s is the pre-US score. The mice were then allowed to explore again for 1 min, and the auditory cue and shock were again presented, followed by another 2 min exploration (post-US score). On day 3 (24 hours after training), mice were returned to the same context in which training occurred, and freezing behavior was recorded for 5 min (context test). Ninety min later, freezing was recorded in a novel environment (the grid floor was hidden and a scent of peppermint was added) for 3 min without the auditory cue stimulus (pre-CS test). Finally, the auditory cue was turned on, and the time spent freezing was recorded over the following 3 min (cue CS test).

Spatial learning and memory were examined in a Morris water maze [69,70], which consisted of a circular tank (32.5 cm high × 150 cm diameter), filled with water (up to 16 cm deep), maintained at 26°C, and made opaque with nontoxic white paint. A circular platform (15 cm high × 15 cm diameter) remained hidden 1 cm below the water surface at a fixed position. The room housing the tank had a permanent display of distal extra-maze cues. The swim paths of the mice were recorded using computerized EthoVision video tracking equipment. During training (acquisition phase), the mice were given four swim trials daily with an inter-trial interval of 15 min. The mice were placed in the pool facing the wall at one of four starting positions. If the animal did not find the platform after 120 s, it was guided there by the experimenter. Mice were allowed to rest 15 s on the platform before being removed from the pool. Latency to reach the platform, path length, and average swim speed were recorded. After five training days, there were two days of rest, followed by another five days of training and two days of rest. Probe trials were performed on days 8 and 15. During probe trials, the platform was removed and each animal was monitored once for 100 s, recording the percentage time in each quadrant. Over all the trials, one *Survivin*^*Camcre *^mouse floated with a speed of < 5 cm/s, and this mouse was therefore excluded from the study.

### Statistical analyses

Data are presented as the mean ± SEM. Data were analyzed with a two tailed t-test, Mann-Whitney Rank Sum Test, one way ANOVA, or two way repeated measures ANOVA. All statistical tests were performed at a significance level of 0.05.

## Authors' contributions

VC was involved in designing and performing all experiments. VC, DL, AE, RD'H, UB, MC, VB and HC helped in drafting the manuscript. VC, VR, AD, JC, MM and TJ prepared riboprobes, did cDNA cloning and sequencing, *in situ *hybridizations, *in vivo *studies, histologic sectioning, acquisition of data and analyses. TA, DB, RD'H helped in behavioral studies. FA, YB, MC helped in seizure studies. AE and DL provided continuous intellectual input, evaluation and interpretation of data. EC conceived, designed and co-ordinated the project, and drafted the manuscript. All authors read and approved the final manuscript.

## Supplementary Material

Additional file 1 **Supplemental Figures S1-S6**. **Supplemental Figure S1: CAMKIIα-cre activity in neurogenic regions of embryo**. CAMKIIα-cre recombinase activity in the embryonic brain was checked by breeding CAMKIIα-cre mice with ROSA26-stop-YFP reporter mice. GFP stained coronal section through the ganglionic eminence and dorsal telencephalon of E12.5 CAMKIIα-cre+/-:ROSA26-stop-YFP/wt mice reveals prominent CAM-cre activity in the ganglionic eminences, but less in the dorsal telencephalon. *Survivin *mRNA expression is shown in adjacent section. Scale bars 500 μm. HP, hippocampus; GE, ganglionic eminence; NCX, neocortex. **Supplemental Figure S2: CAMKIIα-cre is not expressed in SGZ or SVZ postnatally**. Sagittal sections through the dentate gyrus (A, C-E) and lateral ventricle (B, F-H) of CAMKIIα-cre+/- adult mouse brain (6 weeks). (A, B) Staining for cre recombinase (red) and DAPI nuclear staining (blue) shows that cre expression is present in the dentate granule cell layer (GCL), the striatum (ST) and the cortex (CTX). Lack of red staining of DAPI+ nuclei in the SGZ and SVZ/RMS confirms that CAMKIIα-cre is not expressed in the SGZ or SVZ NPCs postnatally. Double stainining of the dentate gyrus (C-E) and the SVZ (F-H) for cre recombinase (red) and mature neuronal marker NeuN (green), with overlay of fields (E and H), confirms that CAMKIIα-cre expression colocalizes 100% with NeuN and is not present in SGZ or SVZ NPCs. LV, lateral ventricle. **Supplemental Figure S3: Exogenous gene delivery of survivin in embryonic NPCs may increase OB neurogenesis**. GFP labeling of sagittal sections through the olfactory bulb (OB) of P21 control (A, B) and *Survivin*^*Camcre *^(ko) (C, D) mice that were injected in the cerebral ventricle at E12.5 with control-GFP (A, C) or survivin-GFP (B, D) lentiviral vector. Injection of survivin results in an increased number of embryonic NPC-derived cells in the OB. Scale bars: 500 μm. **Supplemental Figure S4: Cage activity recordings**. Cage activity was recorded at 30 min intervals over 23 hours, monitoring the number of laser beam crossings by each mouse (n = 12 per group). There were no significant differences between control (wt) and *Survivin*^*Camcre *^(ko) mice in the total number of beam crossings (p = 0.40) and no alterations in circadian activity profiles (p = 0.30). Results reflect means + SEM. **Supplemental Figure S5: Visual evoked potentials**. Visual evoked potentail (VEP) recordings from control (wt) and *Survivin*^*Camcre *^(ko) mice, reveal similar peak latency and amplitude for both genotypes. **Supplemental Figure S6: Passive avoidance studies**. The *Survivin*^*Camcre *^mice (ko) exhibited a significant impairment in passive avoidance learning, indicated by their shorter latency to enter the dark compartment than the controls (wt). Click here for file

Additional file 2 **Supplemental Methods and Results**. Additional methods and results Click here for file
