# Supplementary material for: Impaired neurogenesis, learning and memory and low seizure threshold associated with loss of neural precursor cell survivin
Source: BMC Neurosci. 2010 Jan 5;11:2. doi: 10.1186/1471-2202-11-2 (PMC2817683; doi:10.1186/1471-2202-11-2)
Supplement: Additional file 1 — Supplemental Figures S1-S6. Supplemental Figure S1: CAMKIIα-cre activity in neurogenic regions of embryo. CAMKIIα-cre recombinase activity in the embryonic brain was checked by breeding CAMKIIα-cre mice with ROSA26-stop-YFP reporter mice. GFP stained coronal section through the ganglionic eminence and dorsal telencephalon of E12.5 CAMKIIα-cre+/-:ROSA26-stop-YFP/wt mice reveals prominent CAM-cre activity in the ganglionic eminences, but less in the dorsal telencephalon. Survivin mRNA expression is shown in adjacent section. Scale bars 500 μm. HP, hippocampus; GE, ganglionic eminence; NCX, neocortex. Supplemental Figure S2: CAMKIIα-cre is not expressed in SGZ or SVZ postnatally. Sagittal sections through the dentate gyrus (A, C-E) and lateral ventricle (B, F-H) of CAMKIIα-cre+/- adult mouse brain (6 weeks). (A, B) Staining for cre recombinase (red) and DAPI nuclear staining (blue) shows that cre expression is present in the dentate granule cell layer (GCL), the striatum (ST) and the cortex (CTX). Lack of red staining of DAPI+ nuclei in the SGZ and SVZ/RMS confirms that CAMKIIα-cre is not expressed in the SGZ or SVZ NPCs postnatally. Double stainining of the dentate gyrus (C-E) and the SVZ (F-H) for cre recombinase (red) and mature neuronal marker NeuN (green), with overlay of fields (E and H), confirms that CAMKIIα-cre expression colocalizes 100% with NeuN and is not present in SGZ or SVZ NPCs. LV, lateral ventricle. Supplemental Figure S3: Exogenous gene delivery of survivin in embryonic NPCs may increase OB neurogenesis. GFP labeling of sagittal sections through the olfactory bulb (OB) of P21 control (A, B) and SurvivinCamcre (ko) (C, D) mice that were injected in the cerebral ventricle at E12.5 with control-GFP (A, C) or survivin-GFP (B, D) lentiviral vector. Injection of survivin results in an increased number of embryonic NPC-derived cells in the OB. Scale bars: 500 μm. Supplemental Figure S4: Cage activity recordings. Cage activity was recorded at 30 min interva [file 1471-2202-11-2-S1.PPTX]

## Slide 1
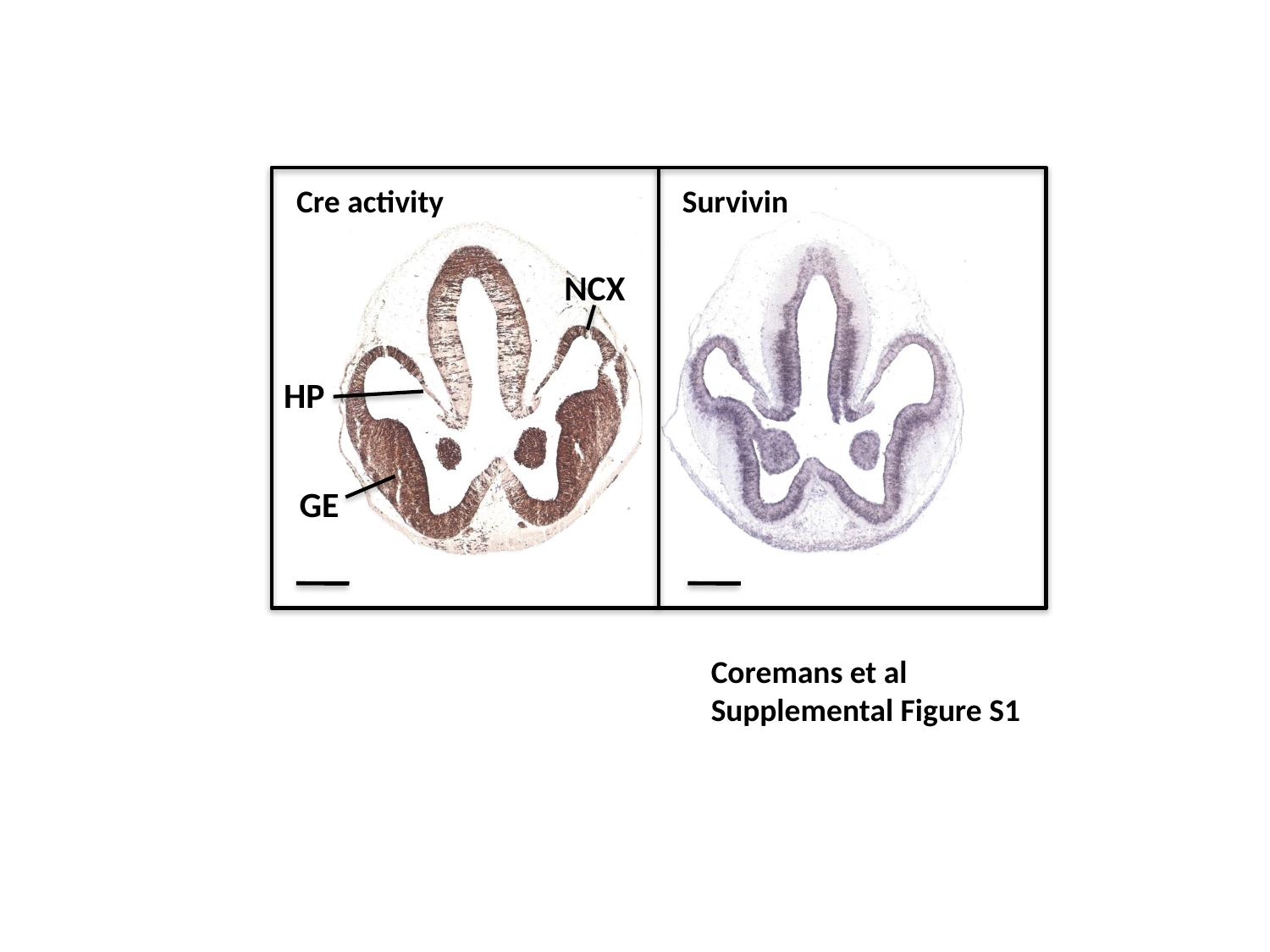

Cre activity
Survivin
NCX
HP
GE
Coremans et al
Supplemental Figure S1

## Slide 2
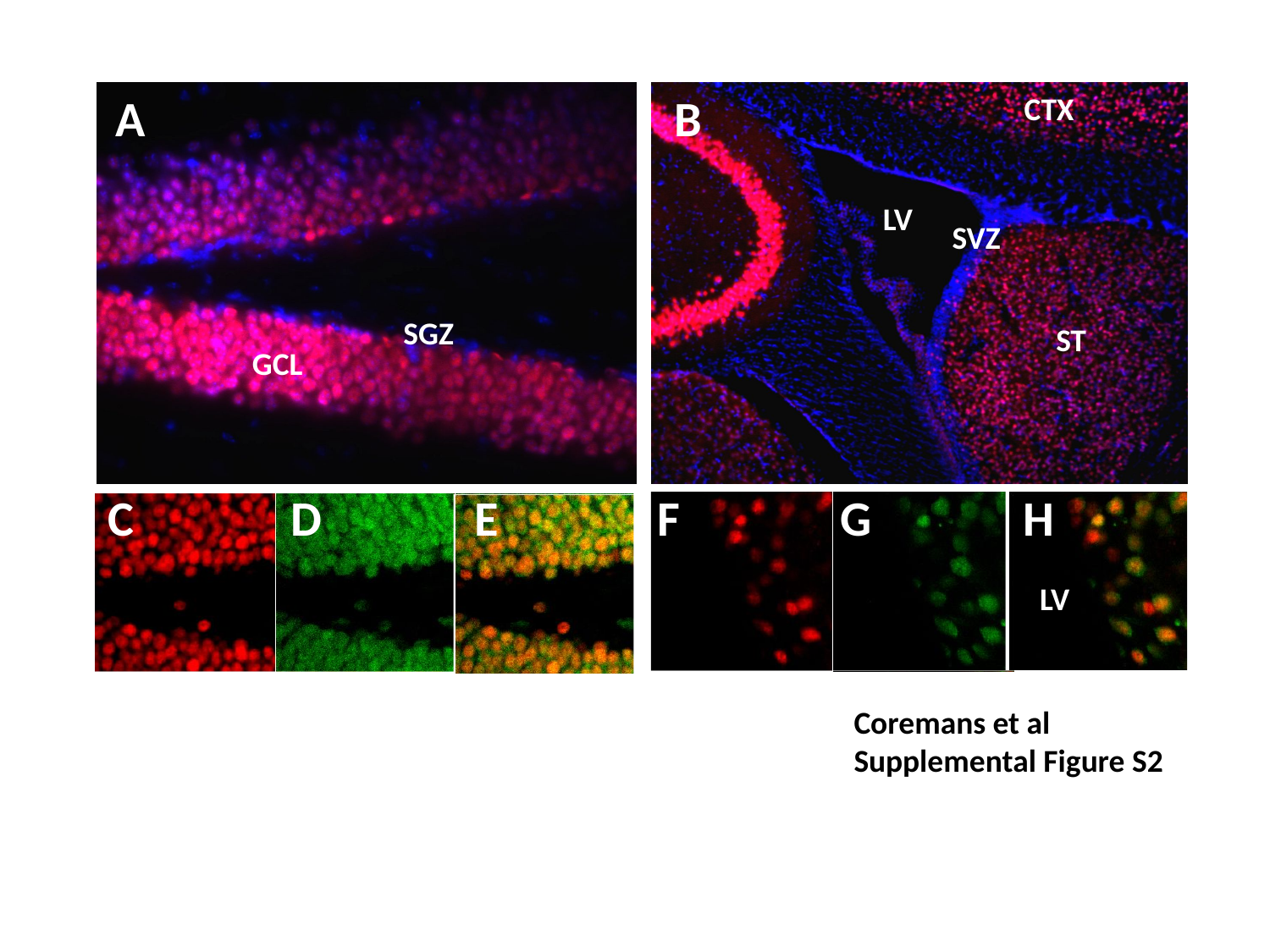

A
SGZ
GCL
B
CTX
LV
SVZ
ST
C
D
E
F
G
H
LV
Coremans et al
Supplemental Figure S2

## Slide 3
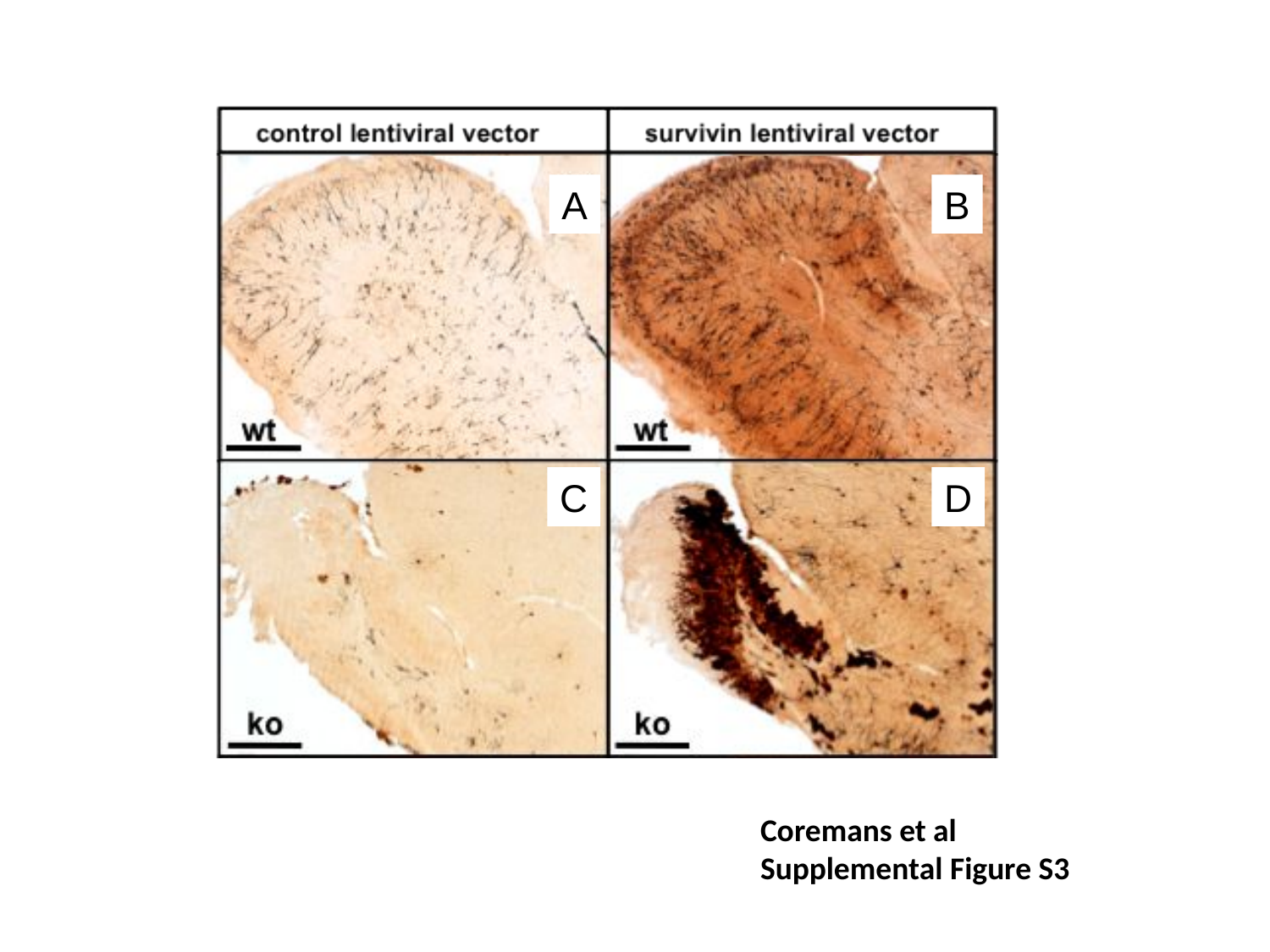

A
B
C
D
Coremans et al
Supplemental Figure S3

## Slide 4
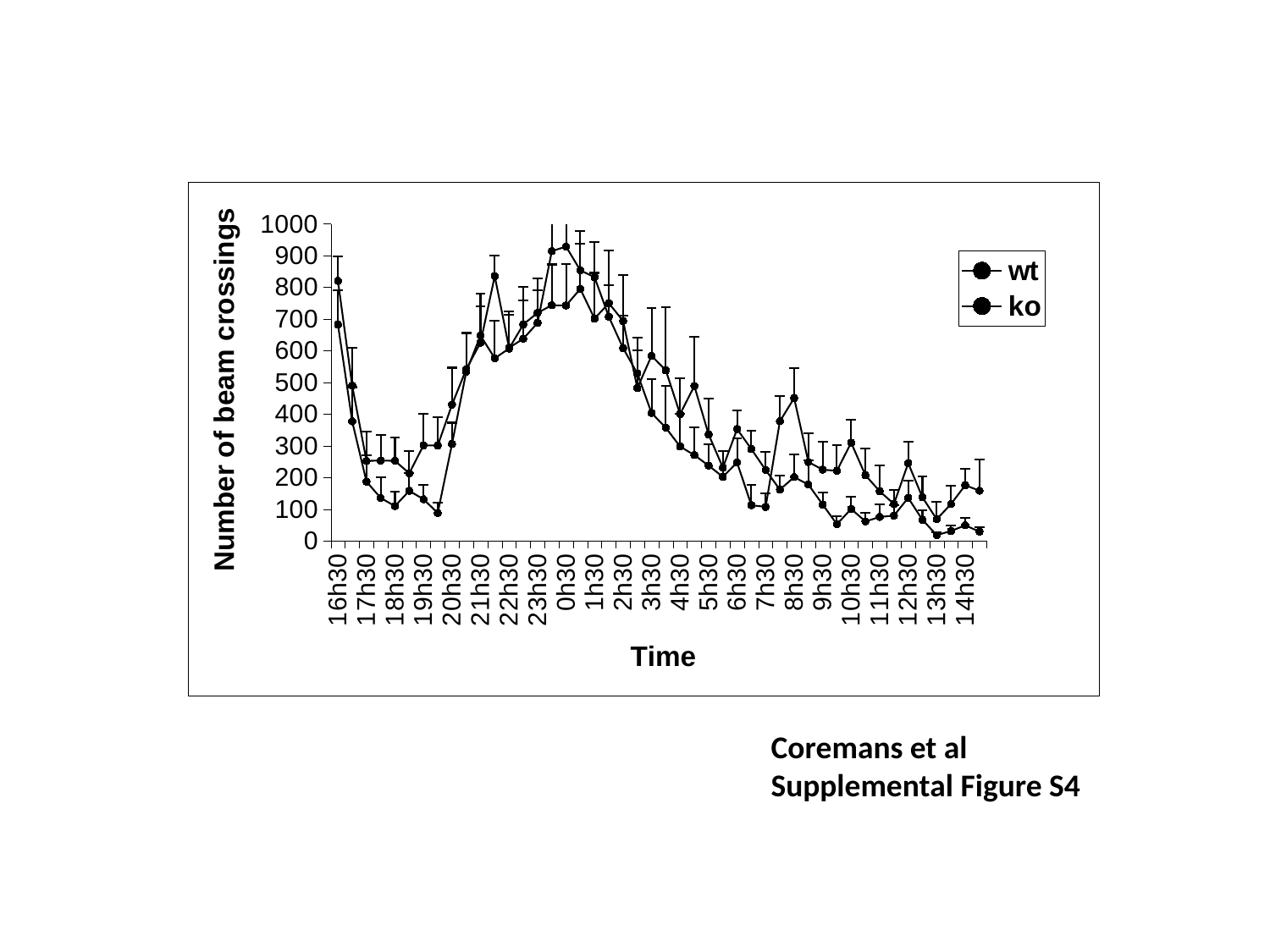

### Chart
| Category | | |
|---|---|---|
| 16h30 | 820.583 | 683.0 |
| 17h00 | 490.083 | 377.462 |
| 17h30 | 252.583 | 187.077 |
| 18h00 | 253.667 | 135.769 |
| 18h30 | 252.833 | 109.923 |
| 19h00 | 213.583 | 158.154 |
| 19h30 | 301.75 | 131.615 |
| 20h00 | 300.917 | 88.308 |
| 20h30 | 429.917 | 305.9229999999989 |
| 21h00 | 542.0 | 534.0 |
| 21h30 | 625.583 | 648.0 |
| 22h00 | 835.833 | 576.615 |
| 22h30 | 609.4169999999984 | 606.8459999999982 |
| 23h00 | 638.0 | 683.308 |
| 23h30 | 688.083 | 719.769 |
| 0h00 | 914.4169999999984 | 743.769 |
| 0h30 | 928.833 | 743.154 |
| 1h00 | 854.0 | 795.308 |
| 1h30 | 832.333 | 701.615 |
| 2h00 | 707.4169999999984 | 750.231 |
| 2h30 | 608.5 | 694.154 |
| 3h00 | 528.5 | 482.462 |
| 3h30 | 403.667 | 584.385 |
| 4h00 | 357.167 | 538.231 |
| 4h30 | 297.833 | 400.385 |
| 5h00 | 271.083 | 489.2309999999989 |
| 5h30 | 237.75 | 336.2309999999989 |
| 6h00 | 202.417 | 231.385 |
| 6h30 | 247.583 | 353.154 |
| 7h00 | 112.917 | 289.9229999999989 |
| 7h30 | 107.333 | 223.923 |
| 8h00 | 377.75 | 162.077 |
| 8h30 | 450.833 | 201.538 |
| 9h00 | 249.083 | 177.846 |
| 9h30 | 224.917 | 114.846 |
| 10h00 | 221.583 | 52.923 |
| 10h30 | 310.0 | 100.846 |
| 11h00 | 207.417 | 61.692 |
| 11h30 | 156.75 | 75.769 |
| 12h00 | 116.417 | 80.0 |
| 12h30 | 245.583 | 136.0 |
| 13h00 | 137.667 | 66.769 |
| 13h30 | 68.667 | 18.538 |
| 14h00 | 116.25 | 31.385 |
| 14h30 | 176.0 | 49.308 |
| 15h00 | 158.417 | 29.385 |Coremans et al
Supplemental Figure S4

## Slide 5
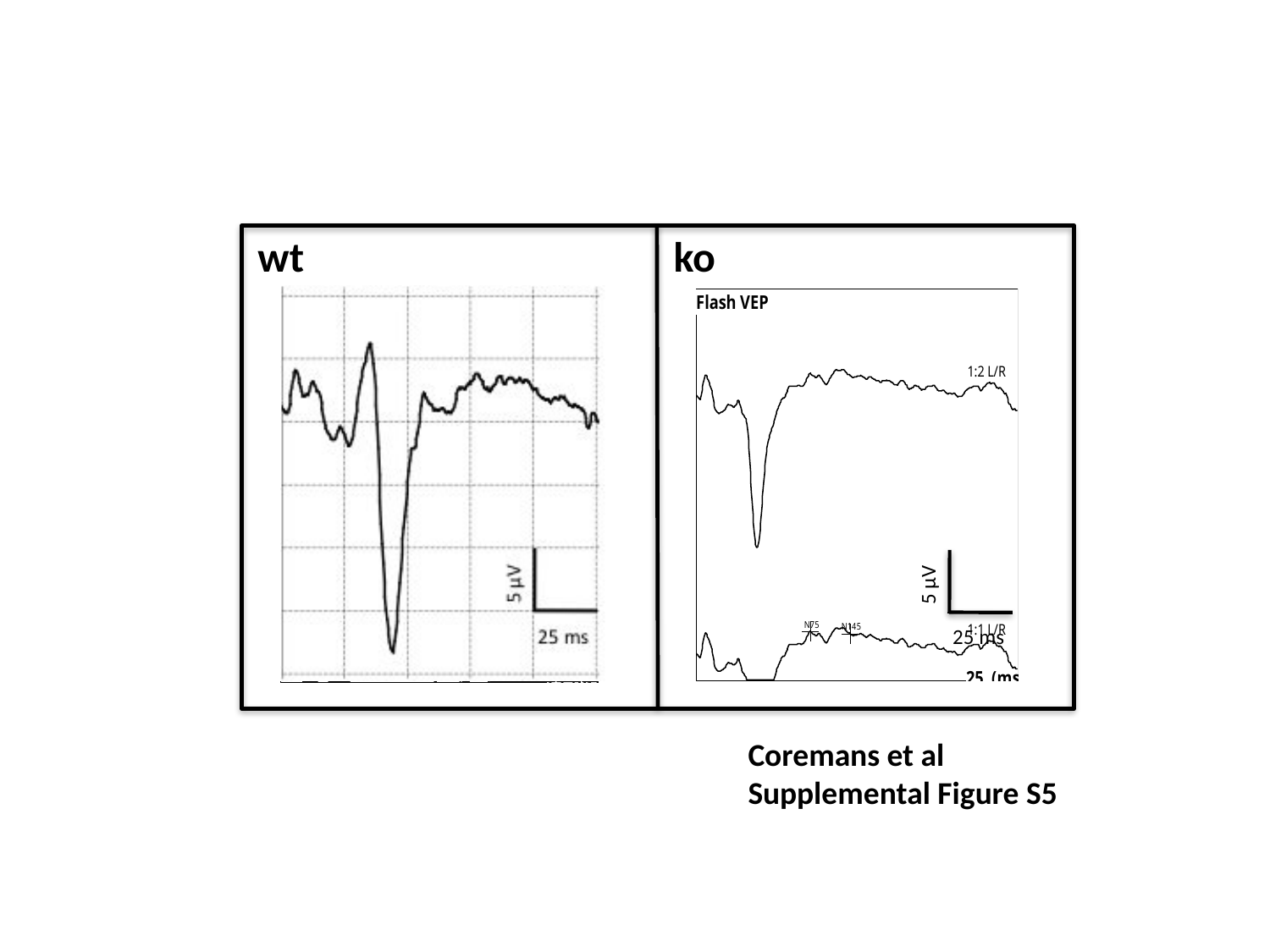

wt
ko
5 μV
25 ms
Coremans et al
Supplemental Figure S5

## Slide 6
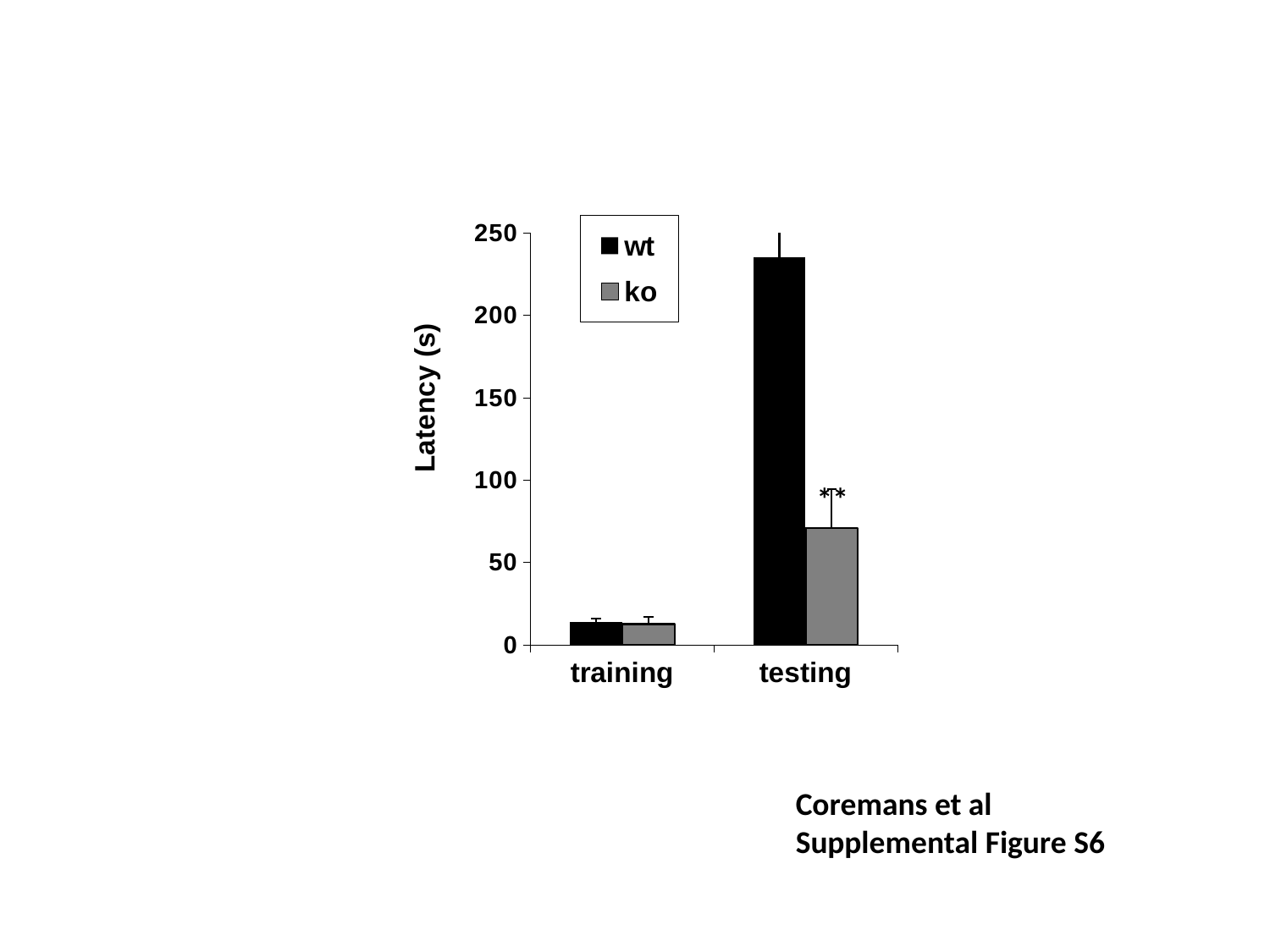

### Chart
| Category | | |
|---|---|---|
| training | 13.917 | 12.538 |
| testing | 235.25 | 70.846 |**
Coremans et al
Supplemental Figure S6
