# Supplementary material for: Impaired neurogenesis, learning and memory and low seizure threshold associated with loss of neural precursor cell survivin
Source: BMC Neurosci. 2010 Jan 5;11:2. doi: 10.1186/1471-2202-11-2 (PMC2817683; doi:10.1186/1471-2202-11-2)
Supplement: Additional file 2 — Supplemental Methods and Results. Additional methods and results [file 1471-2202-11-2-S2.rtf]

Additional File 2
Supplemental Methods
Grip strength was measured using a T-shaped bar connected to a digital dynamometer (Ugo Basile, Comerio, Italy).  The mouse was lifted by the tail so that its forepaws were allowed to grasp onto the bar, and gently pulled backward until the grip was released. Ten measurements were recorded per animal (n=12 mice per group). Motor coordination and balance were tested on an accelerating rotarod (MED Associates, St.Albans, Vermont, USA). After training at constant speed (4 rpm, 2 min), four trials with an inter-trial interval of 5 min were performed. The animals were placed on a rotating drum that accelerated from 4 to 40 rpm over a 5 min period, and the latency to fall from the rod was recorded up to 300s cut-off. 
The tail flick test was used to measure pain threshold to confirm that there are no genotype-dependent differences in sensitivity to the shocks that are administered in several of the behavioral tasks. Three cm of the mouse tail was immersed into a 52°C water bath and the amount of time until the mouse flicked or removed its tail was recorded.
Circadian cage activity was assessed using a transparent cage (20 cm x 26 cm) placed between three infrared beams, which were connected to a lab-built activity logger. The mice (n=12 mice per group) were placed individually in the cage for 23 hours, during which time beam crossings, representing activity, were counted for each 30-min interval.
Visual evoked potentials (VEP) and brainstem auditory evoked potentials (BAEP) were recorded on a Myos 4 plus digital EMG/EP recorder (Schwarzer, Munich, Germany) using subcutaneous platinum needle electrodes during pentobarbital anesthesia to check if mice have normal visual and auditory skills. For VEP recordings, one electrode was placed between the eyes, and a reference electrode and a ground lead were placed at the base of the skull and tail, respectively. Two hundred flash stimuli were presented, and the responses were averaged. For BAEP recordings, one electrode was placed above each ear and referenced to a common electrode near the base of the tail. A fourth electrode, also placed near the base of the tail, was used as ground. Tracings were obtained by averaging 2000 responses evoked by 85-86 dB clicks emitted by a speaker placed 1 cm in front of the animal's head.  

Supplemental Results
Sensory and neuromotor performance
Grip strength measures were not different between groups (n=12 mice per group, p>0.05).  Nor was there was a difference in accelerating rotarod performance (n=12 mice per group, p=0.066).
The tail flick test confirmed that there was no difference in acute pain treshold between control and SurvivinCamcre mice (tail flick latency = 2.9 + 0.7 and 4.4 +1.2 sec, respectively; n=12 mice per group, p=0.25).  
During the 23 hour cage activity recordings, there were no differences in the total number of beam crossings (n=12 mice per group, p=0.40) and recordings for each 30-min interval over the 23 hours revealed no alterations in circadian activity profiles (n=12 mice per group, p= 0.30) (Additional file 1: Supplemental Figure S4).
Recordings from visual evoked potentials (VEP) did not reveal a difference in the latency of the main negative peak: 53.2 + 1.5  and 54.5 + 1.4 ms for control (n=11) and SurvivinCamcre mice (n=10), respectively (p=0.52) with similar amplitude for both genotypes (Additional file 1: Supplemental Figure S5). Both control and SurvivinCamcre mice displayed 5-peak responses on the BAEP tracings that were of similar amplitude. These data indicate that the visual and auditory sensitivity in the SurvivinCamcre mice was not different from controls.
